# Exploratory Puncture of the Drum Membrane in Certain Diseases of the Middle Ear

**Published:** 1907-06

**Authors:** Dunbar Roy

**Affiliations:** Atlanta, Ga.


### Journal-Record of Medicine

Successor to Atlanta Medical and Surgical Journal, Established 1855, and Southern Medical Record, Established 1870.

OWNED BY THE ATLANTA MEDICAL JOURNAL CO.

Published Monthly.

*Official Organ Fulton County Medical Society, State Examining Board, Presbyterian Hospital, Etc.*

VOL. IX. JUNE, 1907. No. 3

BERNARD WOLFF, M. D., EDITOR, 319-320 Prudential Bldg.

GEORGE BROWN, M. D., BUSINESS MANAGER, Business Office 1½-5½ S. Broad St.

E. G. BALLENGER, M. D., ASSOCIATE EDITOR AND ASSISTANT BUSINESS MANAGER.

### ORIGINAL COMMUNICATIONS.

### EXPLORATORY PUNCTURE OF THE DRUM MEMBRANE IN CERTAIN DISEASES OF THE MIDDLE EAR.*

By Dunbar Roy, M.D., Atlanta, Ga.

To the medical student there is no literature so interesting as that which deals with the report of cases. As a teacher I have frequently tried to solve the reason for this. If one will but consider for a moment, he will readily discover wherein lies this peculiarity. I never read the report of a case in my own line of work but that it brings up another which has occurred in my own practice wherein similar or dissimilar characteristics are frequently seen.

I remember with what pleasure I used to peruse the pages of that old work of Toynbee's upon the ear and even now I read the same with pleasure and profit, all because he illustrates every subject with a number of cases which had occurred in his own practice.

To me such reports are exceedingly interesting and instructive, so much so that I am free to admit that the practical report of unusual cases carries more information with it than pages of theoretical suggestions. Such reports appeal to you as incidents which have actually occurred and which may occur at any time in your own practice. It is for this reason that I report to-day the histories of two cases and from them draw some deductions as bearing upon the title of this paper.

While many of you present may have had similar cases and many have had no difficulty in their diagnosis and treatment, the fact is true, however, that the various text-books on otology are silent upon such possible conditions being present during the course of a middle ear trouble and even other literature at my command is silent upon the subject.

Miss B., age 40, consulted me in June, 1899, on account of deafness, noises in the head, and intense pain around the ear.

From her mother I obtained the following history: Patient was never strong and robust. Was of a nervous temperament and two years ago was treated by a prominent neurologist for what he diagnosed as hysterical insanity. She had never been troubled with her ears up to five months ago when she was seized with severe pain in the left ear. She was treated by her home physician with practically no result. On April 10, one month later, she came to Atlanta and placed herself under the care of an otologist. He told her she was threatened with a mastoid abscess. The drum was punctured but she said she never felt or saw any discharge. She remained under treatment until June, and then returned home still suffering with pain. On June 26th she consulted me.

On examination I found a fairly vigorous looking woman of small stature. Wears glasses for hyperopia. Has a saddle back nose due to a perforated septum. She gives no history of syphilis, and the perforation was due to a traumatism. The mucous membrane of the nose looks healthy. Has some posterior hypertrophies of both inferior turbinates. Naso-pharynx shows nothing abnormal.

Right ear is normal in appearance and hearing power.

Left Ear: Canal perfectly clear and no congestion present at any point. The drum appears thickened and opaque but absolutely no redness. By inflation, the eustachian tube appeared well open. No swelling or tenderness even on deep pressure over the mastoid.

Hearing: Watch 6 inches. Whisper, one yard.

T. F. of middle register shows B. C. much prolonged over A. C. Same relationship, holds with forks of all registers. Complains of intense pain in front of ear and in the occipital region. A provisional diagnosis was made of otalgia and hysterical neuralgia. Patient was put upon increasing doses of iodide of potassium. Iodine was painted over the mastoid. The galvanic current was applied to the neck and occipital region. Menthol and iodine were passed per catheter into the middle ear.

At 12:30 the same night of the first examination, I was called by telephone to see the patient. She was complaining of intense pain in the occipital region. This was relieved with an anodyne and the hot water bag. The next day the patient was as comfortable as on the day previous. The line of treatment commenced was continued with a varying degree of success until June 30th, when the pain continuing with such severity I decided to incise the drum membrane as an exploratory measure.

This was done under thorough antiseptic precautions, the incision being made posteriorly and well up into Shrapnell's membrane. This was followed by some blood and glairy mucus. The patient immediately experienced a feeling of relief. She was instructed to syringe the ear with hot carbolized solution every three hours.

The next day the patient still felt much relieved and the opening made continued patulous. Suction was made upon the drum with a Siegel's speculum during which there appeared at the incision a small red body like a polypus. This was seized with a pair of forceps and removed, it proving to be a mucous polypus about the size of a pea. From this time on the patient rapidly improved and in a few days the discharge ceased, the incision in the drum healed, all pain disappeared and the hearing power was equal to that in the other ear.

This case is reported in detail because its diagnosis was veiled in some obscurity. Since this observation and from further practical proof obtained in other cases, I am convinced that the majority of workers in otology reverence too sacredly the idea of puncturing the drum membrane. I do not believe that the puncturing of the drum membrane should be done rashly and at random, but I do believe that if we are convinced of a middle ear trouble we should not hesitate to puncture the membrana tympani as an exploratory measure.

If this little operation is done under thorough antiseptic precautions, I believe that no harm ever results. These ideas are referable to cases both acute and chronic.

A great deal has been said and written about the puncture of the drum membrane in acute otitis media. Both sides have their advocates and after all such is but an expression of individual experience. He is a poor doctor who does not demonstrate for himself the usefulness of any remedy. Personally, I believe that I have been able to save my patients many hours of suffering and pain and many days of anxious watching and waiting by an early puncture of the drum membrane. I have never seen bad results follow this operation but on the other hand I have seen bad results follow when it was not used. I never hesitate to puncture a drum membrane if it is beefy red in toto and the patient is suffering with a throbbing deep seated pain. In such cases I have never failed to find secretion which, by its pressure presence was the cause of the pain.

Many a mastoid abscess has been aborted by an early and free incision in the membrana tympani.

In cases of subacute otitis media or where an acute case is long drawn out, where the congestion has disappeared from the drum and the ear still feels full and not relieved by inflations, in such cases an exploratory puncture will do no harm and frequently prove of instantaneous relief to the patient.

Years ago Politzer called attention to the fact that frequently great relief followed the puncture of the drum where the same was greatly retracted, and there were marked symptoms of tinnitus and fullness in the head. In fact, in his 1893 edition he devotes a chapter to this subject and lays down five indications for this procedure:

1. In abnormal thickening and hardening of the drum.

2. In immobilization of the malleus and incus on account of adhesive bands in the tympanic cavity.

3. Where there is a marked impermeable stricture of the eustachian tube.

4. When there are very intense and lasting noises in the ears which have not been relieved by previous ordinary treatment.

5. As preliminary to intratympanic operations.

The incision of the drum membrane under the above conditions has frequently proven to be of great value to me.

As otologists, I think we should try to eradicate from the minds of the laity the prevailing idea that the hearing is destroyed if the drum is punctured.

On the other hand I believe that such a puncture as a therapeutic measure is of much more value than is usually considered.

The second case is one of mastoid involvement following an acute otitis media where all objective symptoms were practically absen..

On May 13th, 1902, I was called by telephone to Macon, Ga., with instructions to come prepared to operate on a probable brain abscess.

I arrived about 8 p. m., and was driven to the home of Rev. L. L. W., the patient, where I met the home physicians in consultation.

From his attending otologist, a most excellent man, I ascertained the following history:

Four weeks ago the patient was visiting in Gainesville, Ga., and while there was attacked with severe pain in his right ear. He immediately returned to his home in Macon to consult an aurist.

His physician gave me the following history: Patient suffering with quite a little pain in the right ear, auditory canal was normal in appearance. The drum membrane was congested throughout but absolutely no bulging. There was no rise in temperature. An acute otitis media was readily diagnosed.

Under the usual treatment all the unpleasant symptoms disappeared. His general health seemed much below par, so his family physician took him in charge.

As he continued to have an occasional rise in temperature and some chilly sensations he was treated for malaria. The patient still complaining of some pain in the ear, the aurist was again consulted. On examination he found a trace of pus in the auditory canal. This was wiped away and it seemed to come from a little spot in the upper part of the canal. The drum was normal in appearance and there was no redness at any point. The patient still continued to suffer pain with occasional exacerbations of fever and some chills. He was given as much as 5 grains of codein daily to relieve his suffering. There was at no time any oedema or pain over the mastoid. In fact, all pain was confined to the vertex of the skull and to the occipital region.

On arrival I found the patient fairly comfortable under the influence of codein. Temperature was 101F. Absolutely no pain could be elicited by pressure over the mastoid. The pain was confined more especially to the side and vertex of the head. The temperature in the morning had been almost normal. The auditory canal showed absolutely no abnormality and in contour it seemed just the same as on the other side. There were no signs of moisture. The drum also appeared perfectly normal and I looked in vain for even a trace of congestion. It had just the appearance of a drum in dry catarrh of the middle ear. As exploratory only, I incised freely the drum membrane. There was an escape of a small amount of blood and muco-pus. Believing that the mastoid was involved, I advised immediate operation. This being accepted, the patient was removed to the City Hospital, where, with the assistance of Drs. Peete, Clark and Moore, the mastoid was opened. This bone was of a very peculiar formation, very prominent and exceedingly convex on the outer surface. Considerably healthy bone had to be passed through before the antrum was reached. Only a drop of pus was found here, but on enlarging the opening several large cells were brought into view which were filled with pus. All of the mastoid was removed to the tip. A free communication was made with the middle ear. The bone over the sinus appeared healthy. The wound was then packed with idoform gauze and dressing applied. A remarkable feature all during the operation, was the profuse muco-purulent discharge through the auditory canal. The patient was put to bed in a very good condition. The next morning at 7 a. m., I called by to see him on my way to the train and found him free from fever and pain and expressing himself as being exceedingly comfortable. He made an uninterrupted recovery, and is now in better health than ever before.

This case presents some peculiarities. In the first place, it illustrates the point which I attempted to make prominent in the report of the first case, and that is the value of an exploratory puncture of the drum membrane.

The history of this case shows that there was absolutely no indication for puncture at any time during the course of the disease. I mean by indications such as are usually laid down by text-books on otology.

The appearance of the drum membrane in this case was almost perfectly normal. The question also naturally arises would not this case have recovered without an operation upon the mastoid had the membrana tympani been incised freely early in the course of the disease?

While an affirmative answer cannot be given with certainty, I believe that the chances for such a result would have been most excellent. I am even free to admit that there was a possible chance for such a termination at the time the puncture was made by me, since there was found to be such a free communication between the mastoid antrum and the middle ear, as indicated by the free discharge through the auditory canal after the puncture was made.

The second feature of this case was the total lack of all objective symptoms of mastoid involvement. There was never any tenderness over the mastoid, even under deep pressure; there were no signs of a sagging of the upper and posterior wall of the canal. The patient had many of the symptoms of malaria and it was very easy to see how a mistaken diagnosis could have been made. In these southern climates, where malaria is frequent, I have seen several cases where it was difficult to make a differential diagnosis. In country places one is not always able to have a blood analysis made.

Cases of this kind but teach us that stereotyped symptoms do not always occur and that we must be prepared for anomalous conditions at any time.

Every case reported, which has features distinctive rom those usually seen, is of value to the profession as adding to the sum total of our knowledge.

DISCUSSION OF DR. ROY'S PAPER.

Dr. M. A. Purse asked if incision was the best method of relieving the pain caused by rapid involvement of the ear in scarlet fever or dyptheria.

Dr. Roy replied that in children these nearly always perforated of themselves within 24 hours, but in adults the membrane is tougher and it is necessary to make an opening for drainage.

